# Readily accessible multifunctional fluorous emulsions†
†Electronic supplementary information (ESI) available: Fig. S1–S4, Scheme S1, detailed experimental procedures, characterization of **27**. See DOI: 10.1039/c6sc00341a


**DOI:** 10.1039/c6sc00341a

**Published:** 2016-04-26

**Authors:** Ellen M. Sletten, Timothy M. Swager

**Affiliations:** a Department of Chemistry , Massachusetts Institute of Technology , 77 Massachusetts Ave , Cambridge , MA 02143 , USA . Email: tswager@mit.edu

## Abstract

Strategies for the facile fabrication of nanoscale materials and devices represent an increasingly important challenge for chemists. Here, we report a simple, one-pot procedure for the formation of perfluorocarbon emulsions with defined functionalization. The fluorous core allows for small molecules containing a fluorous tail to be stabilized inside the emulsions. The emulsions can be formed using a variety of hydrophilic polymers resulting in an array of sizes (90 nm to >1 micron) and surface charges (–95 mV to 65 mV) of fluid particles. The surface of the emulsions can be further functionalized, covalently or non-covalently, through *in situ* or post-emulsion modification. The total preparation time is 30 minutes or less from commercially available reagents without specialized equipment. We envision these emulsions to be applicable to both biological and materials systems.

## Abstract 2

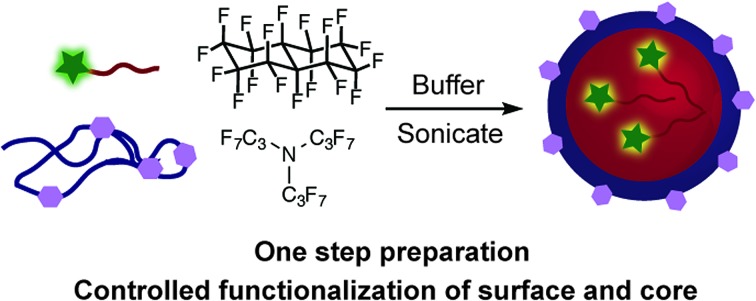
Mixtures of perfluorocarbon and water containing functionalized polymer surfactants and fluorous-tagged small molecules yield multifunctional emulsions with defined functionality on the inside and outside of the droplets.

## Introduction

Over the past decade, significant interest has been placed on nanotechnology as the demand for portable electronics,^1^ mobile diagnostic tools,^2^ and multifunctional therapeutics^3^ increases. At the core of the nanotechnology movement is the discovery of materials that act as building blocks for devices and are readily available to a broad range of scientists. It is the chemists' duty to create nanomaterials that can be easily prepared from commercial reagents and readily customized for use in diverse applications.

**Fig. 1:**
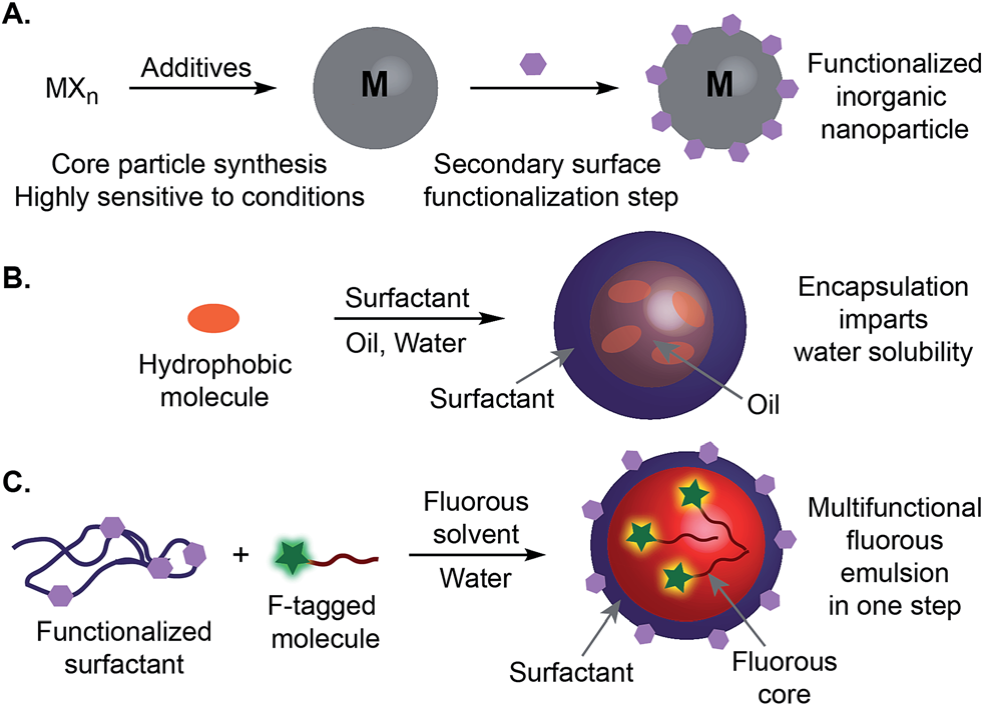
(A) General synthesis of inorganic nanoparticles. First, a metal salt undergoes nucleation to create the nanoparticle core. Specific, controlled conditions allow for different shapes and sizes to be prepared. In a second step, the desired surface functionality is installed. M = metal, X = counterion. (B) Oil-in-water nanoemulsions are used to stabilize and solubilize hydrophobic molecules in aqueous media. (C) Strategy for the one-step preparation of multifunctional fluorous nanoemulsions using a fluorous (F)-tagged molecule, a functionalized surfactant, fluorous solvent, and aqueous media.

Some of the most successful nanomaterials to date are particles.^4^ Nanoparticles have been synthesized from an array of materials with Au, Fe_3_O_4_, and quantum dots being standouts displaying unique, often size-dependent, properties.^5^ These inorganic nanoparticles are prepared in multiple synthetic steps beginning with formation of the core followed by a second chemical transformation to modify the surface for enhanced stability, solubility, and functionality of the materials (Fig. 1A).^6^ These syntheses commonly involve many different reagents and solvent extraction steps, which can be a significant drawback to those not versed in chemical methods and limit the utility of even the most promising materials. Additionally, the use of inorganic nanoparticles, particularly quantum dots, suffers from toxicity concerns, which have prevented their widespread use in biological and environmental devices.^7^ A class of nanoparticles that overcomes both the toxicity concerns and synthetic challenges of inorganic particles are self-assembled organic nanomaterials such as polymer nanoparticles, micelles, vesicles, liposomes, and emulsions.^4^ These dynamic structures are extensively employed in the medical and food industries to protect and solubilize active ingredients in aqueous media (Fig. 1B). Additionally, they are ubiquitous in the paint and ink industries.^8^ However, their expansion into other areas of nanotechnology has been hindered by stability concerns and a limited range of surfactants employed for their formation. Here, we aim to impart additional advantages to organic nanoparticles by diversifying the functionality of perfluorocarbon emulsions in a controlled, yet single-step, procedure.

Our one-step synthesis of functional emulsions is summarized in Fig. 1C. A mixture of fluorous solvent and aqueous buffer is sonicated in the presence of a functionalized polymer surfactant and a fluorous-tagged small molecule to yield emulsions with defined functionalization on the inside and outside of the droplets. Key to our strategy is the use of fluorous solvent as the inner phase of the emulsions, which provides opportunities to direct and control the residence time of small molecules inside the emulsion droplets using fluorous tags^9^ as well as enhances the overall stability of the emulsions.^10^ We also expected the extreme hydrophobicity of fluorocarbons to result in emulsions that are more readily stabilized by a variety of surfactants, an important aspect to broadening the scope of these materials.

Initially, fluorous-in-water emulsions were composed of tetrafluoroethylene (TFE). As early as the 1940s, it was realized that surfactants had an important role in dictating the size and shape of the emulsions, as determined by analysis of the polymerized TFE.^11^ The exceptional properties of poly(tetrafluoroethylene) (PTFE, Teflon™)^12^ resulted in a large body of work regarding aqueous TFE emulsions,^13^ which are often stabilized with fluorinated acids. Many other fluorinated polymers are also synthesized by exploiting fluorous emulsions. However, mounting concerns over the safety of fluorinated small molecule surfactants employed for fluoropolymer synthesis^14^ make new formulations for fluorous emulsions a pressing avenue of research.

Fluorinated surfactants and emulsions also play a large role in the paint and coating industry.^15^ Fluorinated surfactants are more effective at reducing surface tensions of colloidal systems than their organic congeners,^16^ rendering them the molecule of choice for many coatings. The robust properties of fluorinated polymers have also resulted in their use in paints. In particular, poly(vinylidene fluoride) (PVDF) has been found to be an advantageous component in many outdoor paints.^17^

Kinetically stable emulsions of perfluorinated compounds in water can be achieved without employing fluorinated small molecule surfactants. Nanoemulsions composed of a fluorous inner phase stabilized by Pluronic polymers and/or phospholipids were developed in the 1970s due to their use as artificial blood.^18^ The following decades yielded much excitement over these materials and numerous formulations, some of which received clinical approval,^19^ were studied in efforts to enhance oxygen content, stability, and pharmacokinetic properties.^20^ Despite their biomedical potential, the use of fluorous nanoemulsions in medical applications has been limited, with most reports focusing on ^19^F-MRI-based cell-tracking and imaging.^21^ Other notable biological applications include a cellular pH sensor^22^ and the delivery of fluorinated anaesthetics.^23^

## Results and discussion

**Fig. 2:**
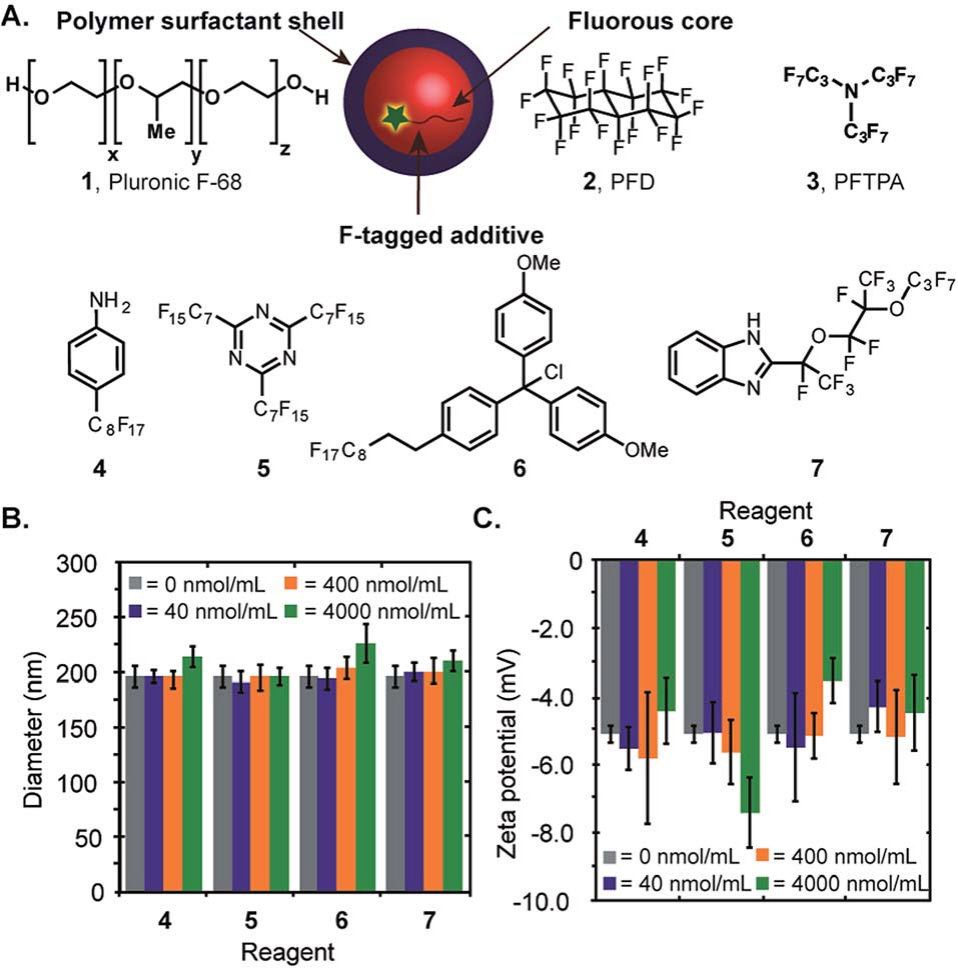
Fluorous-tagged small molecules can be encapsulated inside the nanoemulsions. (A) Schematic of a perfluorocarbon nanoemulsion and the corresponding chemical structures of the surfactant, fluorous solvents, and commercially available fluorous-soluble additives. PFD = perfluorodecalin, PFTPA = perfluorotripropylamine. (B and C) Additives **4–6** were predissolved in a 7 : 3 mixture of PFD/PFTPA at various concentrations and sonicated in the presence of Pluronic-F68 (2.8 wt%) dissolved in PBS. The size (B) and charge (C) of the emulsions were measured. Error bars represent the polydispersity as measured by DLS (B) or the standard deviation of five zeta potential measurements (C).

For demonstration of multifunctional perfluorocarbon nano-emulsions, we employed Pluronic-F68 (**1**) as a surfactant and a 7 : 3 ratio of perfluorodecalin (PFD, **2**)/perfluorotripropylamine (PFTPA, **3**) as the inner phase (Fig. 2A). This formulation represents a simplified version of Fluosol-DA, one of the early perfluorocarbon nanoemulsions approved by the FDA.^24^ Using this preparation, we analysed the scope of small molecules that could be directed to the centre of the nanoemulsions. The fluorous environment of the droplets made for a clear strategy of directing and stabilizing molecules inside the emulsions using fluorous tags. Horvath and Rabai introduced the use of fluorous tags to solubilize catalysts in perfluorinated solvents in 1994.^25^ Since this seminal report, many applications for localizing and purifying small-molecules in the fluorous phase have emerged.^26^ Consequently, compounds with an array of functionalities can be purchased with fluorous tails.^27^ We purchased fluorous-tagged aniline **4** (63 wt% F), triazine **5** (72 wt% F), trityl **6** (41 wt% F), and benzimidazole **7** (57 wt% F) (Fig. 2B), which contain varying degrees of fluorination and incorporated them into our emulsions at three different concentrations by first dissolving **4–7** in a 7 : 3 mixture of PFD/PFTPA and then subjecting these solutions to sonication in an aqueous solution containing 2.8 wt% Pluronic-F68 (**1**). We analysed the size of the resulting emulsions and found that no significant changes in hydrodynamic diameter were evident when loaded with 0.4 μmol mL^–1^ of any of the compounds studied here and only slight changes in diameter were observed for emulsions with higher loadings of 4 μmol mL^–1^ of **4** and **6** (Fig. 2B). These results indicate that ∼10^5^ to 10^6^ molecules can be encapsulated within the droplets without significant changes in size.^28^

An important aspect of our approach is the ability to modify the functionality inside the nanoemulsion droplets without affecting the surface properties. To assay this, we performed zeta potential measurements on the nanoemulsions containing different concentrations of **4–7**. No significant changes in surface charges were observed at concentrations of 0.4 μmol mL^–1^ and lower suggesting the fluorous-tagged compounds were completely localized inside the emulsions (Fig. 2C). However, at the highest concentration tested, 4 μmol mL^–1^, triazine **5** and trityl **6** displayed surface charge differences when compared to particles lacking payloads, thereby indicating an upper threshold for orthogonally modifying the functionality inside and outside of the emulsion droplets. It is interesting to note that for both the size and surface charge changes, we do not see a trend that correlates to fluorine content. Both “light” (less than 40 wt% F), “heavy” (greater than 60 wt% F), and “in between” compounds can all be incorporated into the nanoemulsions.^29^

**Fig. 3:**
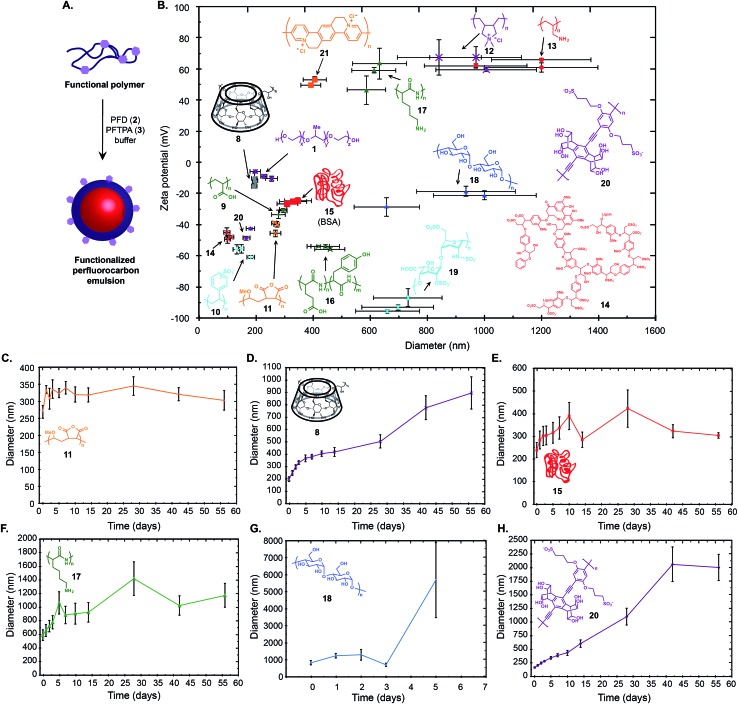
Emulsions with different surface properties can be prepared. (A) Schematic for the simple preparation of functionalized fluorous emulsions. (B) An array of emulsions with different sizes and surface charges was obtained by changing the polymer surfactant. Emulsions were prepared by dissolving the indicated polymer in PBS (2.8 wt%, except for **11** and **21** where solubility limited the polymer to 1.6 wt%) and sonicating in the presence of 7 : 3 PFD/PFTPA (20 wt%). The size and charge of each emulsion was measured in duplicate or triplicate and plotted. The *x*-axis error bars represent polydispersity as measured by DLS. The *y*-axis error bars represent the standard deviation of five zeta potential measurements. (C–H) Stability of selected emulsions over time. Error bars represent polydispersity.

Having demonstrated that molecules can be directed and stabilized inside the perfluorocarbon nanoemulsions, we sought to prepare emulsions with different surface properties. Initial approaches to surface functionalization involving the introduction of amphiphilic small molecules or triblock oligomers containing a fluorous segment to Pluronic-F68 stabilized emulsions were unsuccessful as a result of the poor solubility of the custom surfactant and/or inadequate stability of the resulting emulsions. These results prompted us to look toward changing the polymer surfactant altogether. We envisioned that a variety of functional polymers would stabilize the fluorous emulsions (Fig. 3A). Gratifyingly, a screen of polymers revealed that a vast majority of the hydrophilic polymers investigated promoted emulsion formation to give an array of emulsions with different sizes and surface charges (Fig. 3B). From Fig. 3B, it is clear that the easiest emulsions to prepare are 100–350 nm in diameter with negative zeta potentials (lower left portion of plot) and positively charged emulsions are generally larger in size and display greater polydispersity (upper right portion of plot). Emulsions were stabilized by commercially available hydrophilic polymers (**8–13**), biomolecules (**14–19**), as well as custom conjugated polymers (**20**, **21**).^30^ We analysed the stability of the emulsions over two months, and found that the long-term stability varies considerably (Fig. 3C–H and S1†). Emulsions stabilized by **11**, **15**, **16**, and **17** had reasonable stabilities over time. Emulsions made from **8**, **9**, **14**, **20** displayed steady Ostwald ripening^31^ over two months, while emulsions formed from **10**, **18**, and **21** only remained stable for a few days. The array of emulsions in Fig. 3B shows that for applications where emulsions can be immediately prepared and utilized, the options for obtaining functionalized perfluorocarbon emulsions from commercially available materials are plentiful.

**Fig. 4:**
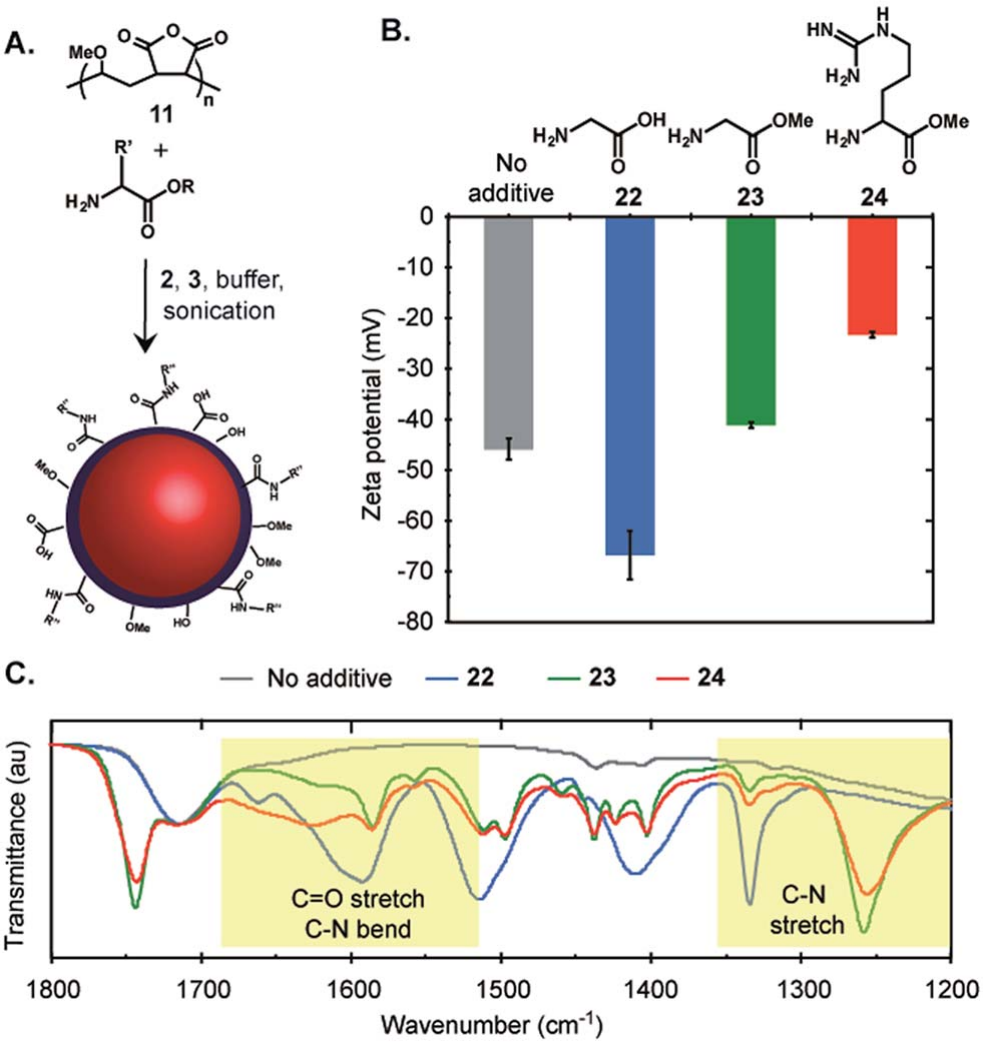
Covalent surface modification of fluorous emulsions. (A) The *in situ* modification of perfluorocarbon emulsions by amines. (B and C) Emulsions were prepared by dissolving 1.6 wt% of partially hydrolysed **11** in PBS containing 200 mM of amine (**22**, **23**, **24**) or as an additive free control and then sonicated in the presence of 20 wt% 7 : 3 PFD/PFTPA. (B) The surface charge of the resulting emulsions was measured. The error bars represent the average of five zeta potential measurements. (C) The emulsions were dried to a polymer residue and analysed by infrared spectroscopy. The spectra were normalized to the dominant carbonyl stretch of **11** at 1712 cm^–1^.

To further customize the surface functionality of the emulsions, we focused on poly(methyl vinyl ether-*alt*-maleic anhydride) (**11**) as a surfactant. If partially hydrolysed prior to emulsion formation, polymer **11** yielded stable, negatively charged emulsions that did not display significant Ostwald ripening over two months (Fig. 3C). We envisioned that the anhydride moiety could be opened by amine nucleophiles *in situ* to modify the surface composition and/or charge of the emulsions (Fig. 4A). This was demonstrated by the addition of glycine (**22**), methyl glycine (**23**), and methyl arginine (**24**) to the mixture of PFD, PFTPA, PBS, and **11** prior to sonication.^32^ We measured the changes in surface charge of the resulting emulsions and found that the emulsions formed in the presence of glycine were significantly more negatively charged (blue, Fig. 4B), methyl arginine were significantly more positively charged (red, Fig. 4B), and methyl glycine were only minimally changed (green, Fig. 4B). To further verify the covalent modification, the emulsions were dried and the remaining polymer surfactants were analysed by infrared spectroscopy (Fig. 4C). New absorbance peaks in the amide bond I and II region (1680 to 1515 cm^–1^)^33^ were observed corresponding to C=O stretches and C–N bends. Additionally, the amide bond III region^34^ from 1350 to 1200 cm^–1^ also exhibited new absorbance peaks resulting from the presence of C–N stretches. Collectively, these data suggest covalent modification of the fluorous emulsion surface. Further support of covalent modification is obtained when analogous experiments are performed with poly(acrylic acid) (**9**) and smaller changes in zeta potential and IR spectra are observed (Fig. S2†). The ability to modify poly(methyl vinyl ether-*alt*-maleic anhydride) nanoemulsions through *in situ* modification allows for facile custom functionalization without additional chemical steps, an advantage over many nanomaterials.

**Fig. 5:**
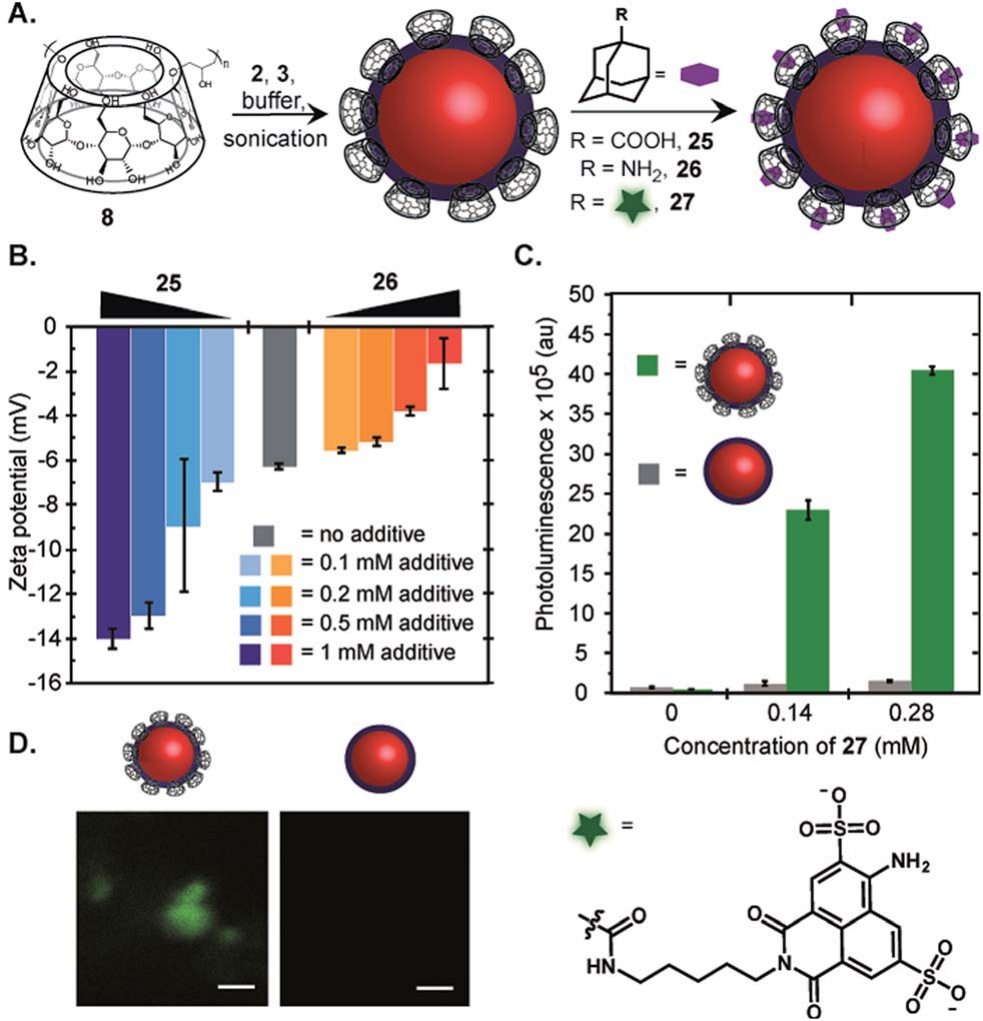
Non-covalent surface modification of fluorous emulsions. (A) Schematic for the modification of perfluorocarbon emulsions with adamantyl compounds. (B) Poly(β-cyclodextrin) emulsions were prepared and adding varying amounts of **25** or **26** in acetonitrile. The acetonitrile was removed by evaporation and the surface charge of the treated emulsions was measured. Error bars represent the average of five zeta potential measurements. (C and D) Poly(β-cyclodextrin) or Pluronic-F68 emulsions were treated with varying amounts of fluorescent adamantyl **27**. The resulting emulsions were thrice washed by gentle centrifugation, removal of the supernatant, and resuspension in PBS. (C) The photoluminescence of the modified fluorous nanoemulsion solutions was measured. Error bars represent the standard deviation of three replicate experiments. (D) Confocal microscopy of poly(β-cyclodextrin) emulsions (left) and Pluronic-F68 emulsions (right) treated with 0.6 mM **27**. Scale bar is 1 μm.

Another simple strategy for modification of the surface is exploiting the affinity of β-cyclodextrin and adamantane (*K*_a_ ∼ 10^5^ M^–1^).^35^ Emulsions stabilized by poly(β-cyclodextrin) (**8**) have size and charge properties similar to Pluronic-F68 nanoemulsions, although they are more prone to Ostwald ripening (Fig. 3D). Nanoemulsions prepared from **8** were subjected to different adamantyl reagents (Fig. 5A). Poly(β-cyclodextrin) stabilized perfluorocarbon nanoemulsions were treated with varying amounts of 1-adamantylamine (**25**) or 1-adamantane carboxylic acid (**26**) and the surface charge was measured (Fig. 5B). A dose-dependent increase in zeta potential with **26** and decrease with **25** suggested successful non-covalent modification of the poly(β-cyclodextrin) stabilized emulsions. Only minimal changes in zeta potential are observed when the analogous experiment is performed with Pluronic-F68 nanoemulsions (Fig. S3†).

To convincingly conclude that non-covalent complexation was a robust strategy for perfluorocarbon emulsion surface functionalization, we conjugated 1-adamantanecarbonyl chloride to Lucifer yellow cadaverine to yield **27** (Fig. 5 and Scheme S1†). Fluorous emulsions formed from poly(β-cyclodextrin) as well as Pluronic-F68 were treated with varying amounts of **27**, thrice washed by centrifugation and resuspension in buffer, and the photoluminescence of the nanoemulsions was measured (Fig. 5C). The poly(β-cyclodextrin) stabilized emulsions displayed dose-dependent fluorescence while the Pluronic-F68 control only displayed minimal background fluorescence. The bulk fluorescence data was confirmed by confocal microscopy (Fig. 5D). We expect the ability to non-covalently modify the surface of perfluorocarbon emulsions will be valuable for elaboration of the surface with sensitive small molecules, biomolecules, or targeting agents that are not amenable to sonication.

**Fig. 6:**
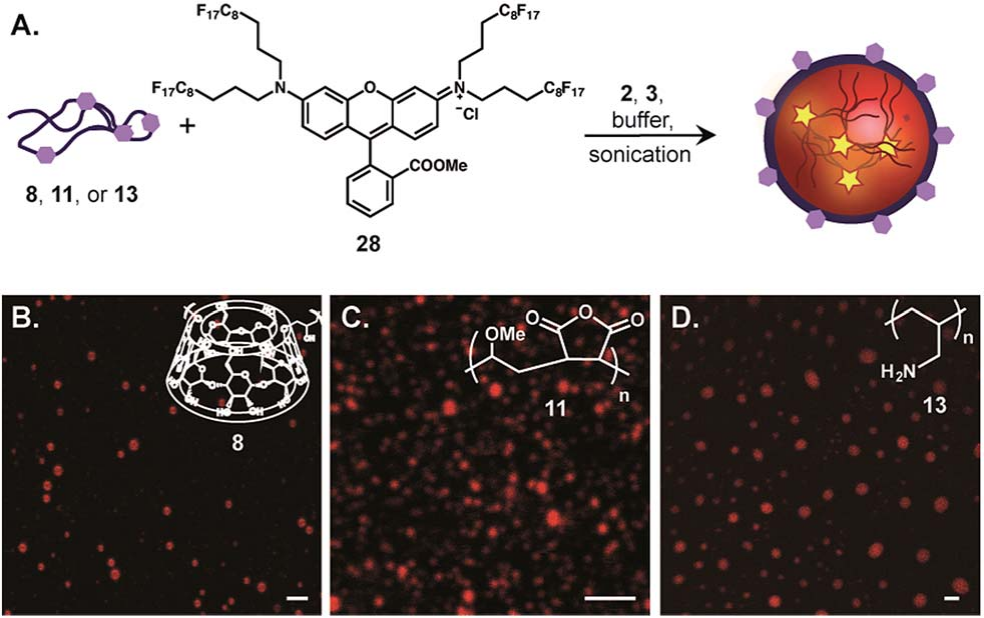
Preparation of multifunctional fluorous emulsions in one step. (A) Schematic for the preparation of perfluorocarbon emulsions containing fluorescent rhodamine **28** on the inside and different outside functionalities. (B) Confocal microscopy of emulsions described in (A). Poly(β-cyclodextrin) (B, **8**, 2.8 wt%), poly(methyl vinyl ether-*alt*-maleic anhydride) (C, **11**, 1.6 wt%), or poly(allylamine) (D, **13**, 2.6 wt%) were dissolved in PBS and sonicated with 7 : 3 PFD/PFTPA. The resulting emulsions were diluted 1 : 100, dropcast onto a clean glass slide, and imaged by confocal microscopy (excitation at 514 nm, collection 530–650 nm). Scale bars represent 2.5 μm (B and C) or 5 μM (D).

Finally, we combined our core and surface functionalization strategies to prepare multifunctional emulsions in one-pot. Emulsions loaded with fluorous rhodamine **28**^36^ and stabilized by poly(β-cyclodextrin) (**8**), poly(methyl vinyl ether-*alt*-maleic anhydride) (**11**), and poly(allylamine) (**13**) were prepared by predissolving **28** in 7 : 3 PFD/PFTPA, introducing the polymer surfactant in PBS and emulsifying *via* sonication (Fig. 6A). The resulting perfluorocarbon emulsions were imaged by confocal microscopy (Fig. 6B–D). As evident from the microscopy, emulsion formation was observed for each polymer surfactant, demonstrating that the properties of the polymer do not influence the ability to encapsulate fluorous-tagged small molecules. Thus, we have devised a generalizable and modular strategy for differentially functionalized emulsion synthesis.

We envision that the multifunctionality and simplicity of emulsion formation will be advantageous for a range of applications. The ability for emulsions to be stabilized by conjugated polymers (*e.g.***20**, **21**) make them of interest to the materials community where processing methods such as spray-on electronics are attractive. Both the surface tension and volatility differences between the fluorous and aqueous systems could be exploited to modulate assembly. Additionally, the fluid nature of the emulsions imparts opportunities for aqueous-based sensing schemes, particularly those involving particle interactions and fusions. Toward this end, the ability to make emulsions with complementary (*e.g.* positive emulsions **12**, **13**, or **17** and negative emulsions **9**, **10**, or **14**) as well as stimuli-responsive (*e.g.* phosphorylation of **16** by kinase, degradation of **19** by heparanase) surface functionality is necessary.

The non-toxic nature of fluorous compounds^37^ coupled with the orthogonality of the fluorous phase provide many opportunities in biotechnology including targeted drug delivery and imaging. We expect emulsions stabilized by anhydride-containing **11** and modified *in situ* with targeting agents, or proteins containing protease-specific cleavage sequences to be of particular interest for these applications. Preliminary results indicate bovine serum albumin (BSA, **15**)-stabilized emulsions undergo trypsin-dependent changes.^38^ Also highly relevant to biomedical applications is the ability to incorporate significant amounts of fluorophore inside the emulsions, which can rival the brightness of quantum dots in aqueous environments using a non-toxic platform.

For some of these applications, the long-term stability and/or size distribution of the emulsions could be a concern. In particular for biomedical applications, small emulsions, that are stable for months are necessary. Our current method to prepare true nanoscale emulsions^39^ is to increase the amount of surfactant; however, with this approach, the smaller the nanoemulsion the more Ostwald ripening occurs (Fig. S4†). Thus, a promising future direction is the development of custom polymers that allow for functionalizable, stable, perfluorocarbon nanoemulsions. Another avenue toward modifying the size/stability of the nanoemulsions is to change the method of emulsification to homogenization or microfluidics.^8^,^40^ The long-term stability of nanoscale emulsions is less imperative for applications in materials science and sensor development. We envision the emulsions reported herein to see immediate use in these areas.

## Conclusions

We have developed methods to differentially functionalize the surface and core of emulsion droplets in one simple procedure. These emulsions can be prepared in ∼20 minutes through simple mixing and sonication of commercially available or custom reagents, depending on the desired application. The choice of fluorous solvent for the inner phase of the emulsions imparts stability and orthogonality in the presence of organic compounds, resulting in enhanced control over the encapsulation and release of small molecules as well as simple surface modification of the emulsions. Work towards the use of customized fluorous emulsions in sensing, bioimaging, and device fabrication is underway.

## Supplementary Material

Supplementary information
