# Supplementary material for: Readily accessible multifunctional fluorous emulsions
Source: Chem Sci. 2016 Apr 26;7(8):5091–7. doi: 10.1039/c6sc00341a (PMC6020120; doi:10.1039/c6sc00341a)

Electronic Supplementary Material (ESI) for Chemical Science.
This journal is © The Royal Society of Chemistry 2016

Supporting Information

for

Readily accessible multifunctional fluorous emulsions

Ellen M. Sletten and Timothy M. Swager

Department of Chemistry, Massachusetts Institute of Technology, Cambridge, MA 02139

Table of Contents

Supporting figures……………………………………………………………………………………S3

Preparation and characterization of emulsions……………………………………………S7

Synthesis and characterization of **27**…………………………………………………………S12

Supporting Figures

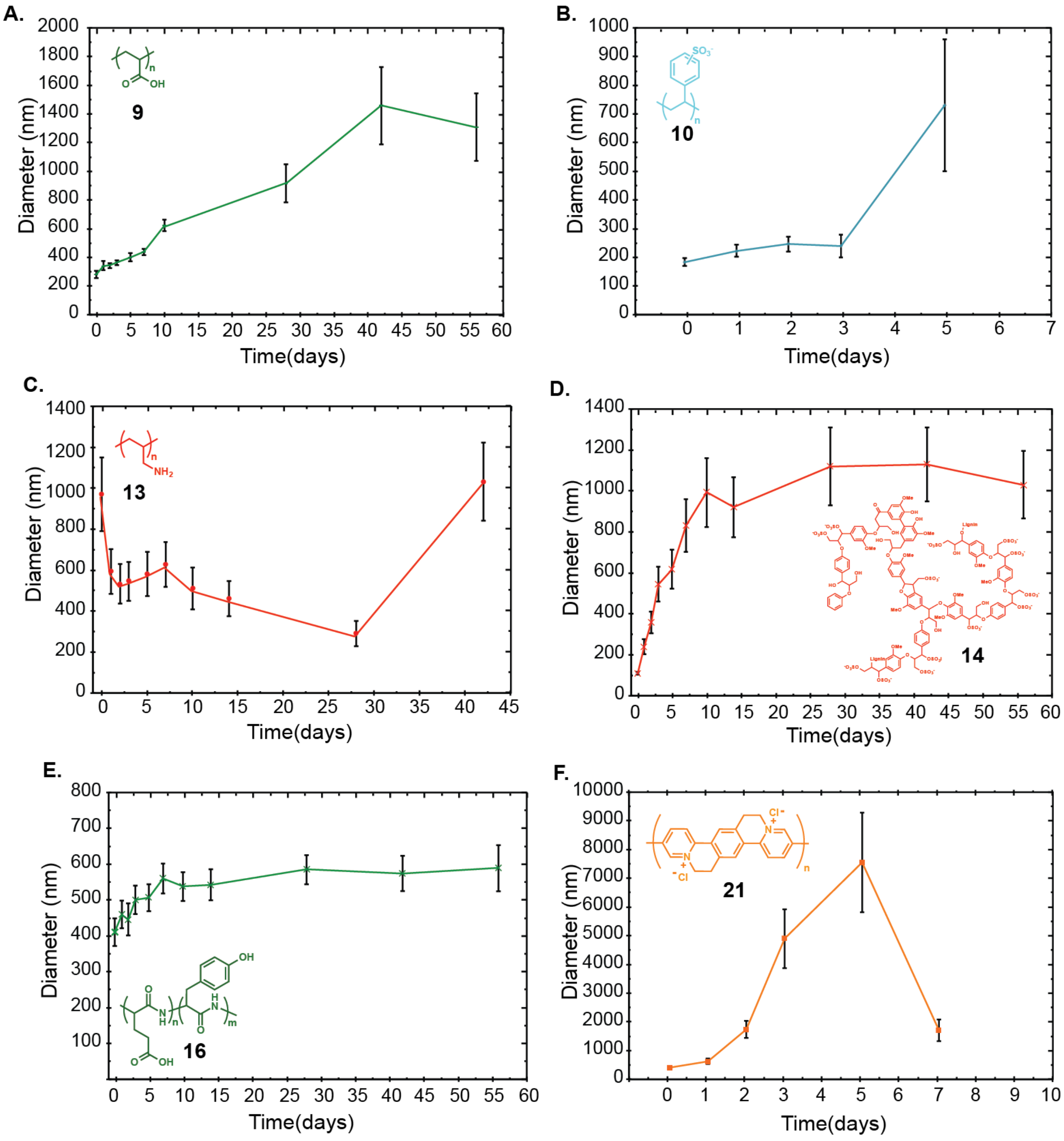

Figure S1. Stability of emulsions prepared from hydrophilic polymers. Emulsions were synthesized by dissolving the indicated polymer in PBS (2.8 wt%, except for **21** where solubility limited the polymer to 1.6 wt%) and sonicating in the presence of 7:3 PFD/PFTPA (20 wt%). Error bars represent the polydispersity as measured by DLS.

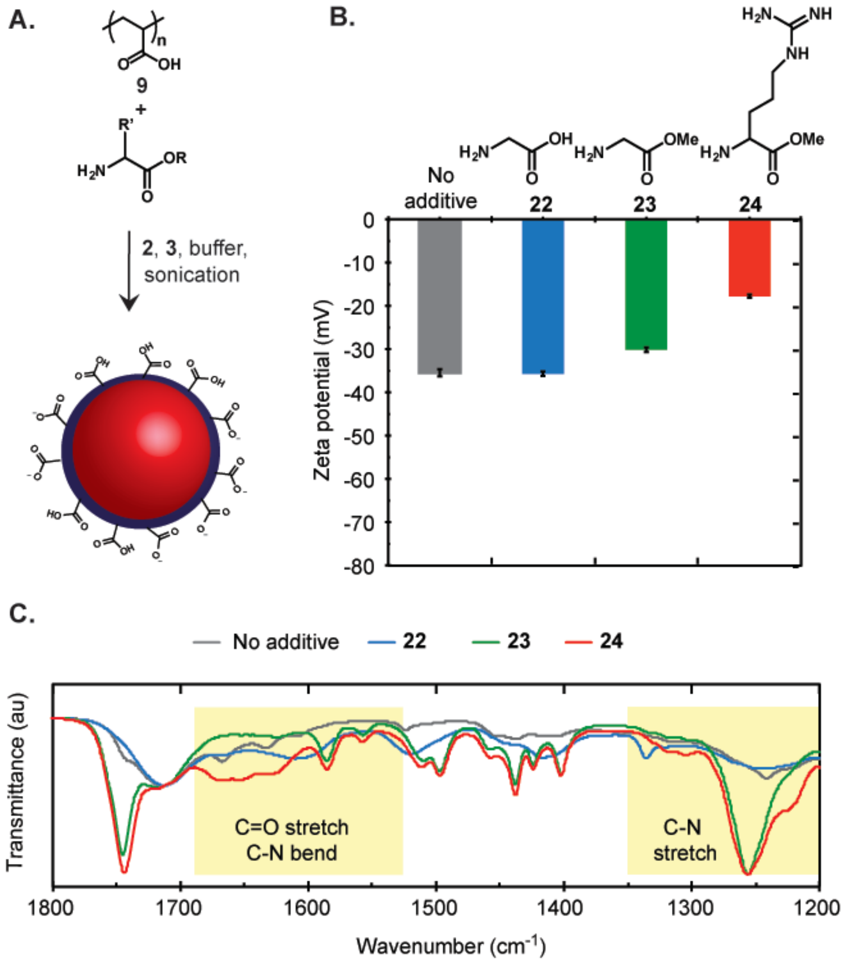

Figure S2. Poly(acrylic acid) control for the modification of perfluorocarbon emulsions with amines. (A) Schematic of the control experiment. (B/C) Emulsions were prepared by dissolving 2.8 wt% of **9** in PBS containing 200 mM of amine (**22**, **23**, **24**) or no additive and sonicated in the presence of 20 wt% 7:3 PFD/PFTPA. (B) The surface charge of the resulting emulsions was measured. Error bars represent the average of five zeta potential measurements. (C) The emulsions were dried to a polymer residue and analyzed by infrared spectroscopy. The spectra were normalized to the dominant carbonyl stretch of **9**.

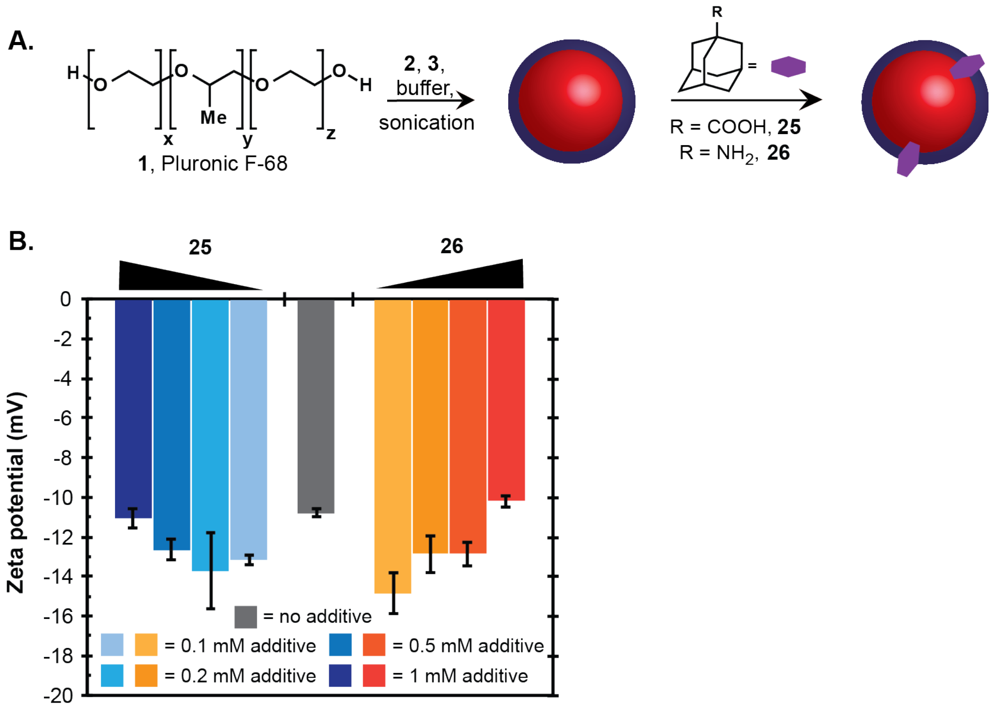

Figure S3. Pluronic-F68 control for the modification of perfluorocarbon emulsions using the adamantane–β-cyclodextrin association. (A) Schematic for the control experiment. (B) Pluronic-F68 nanoemulsions were prepared and varying amounts of **25** or **26** in acetonitrile were added. The acetonitrile was removed by evaporation and the surface charge of the treated nanoemulsions was measured. Error bars represent the average of five zeta potential measurements.

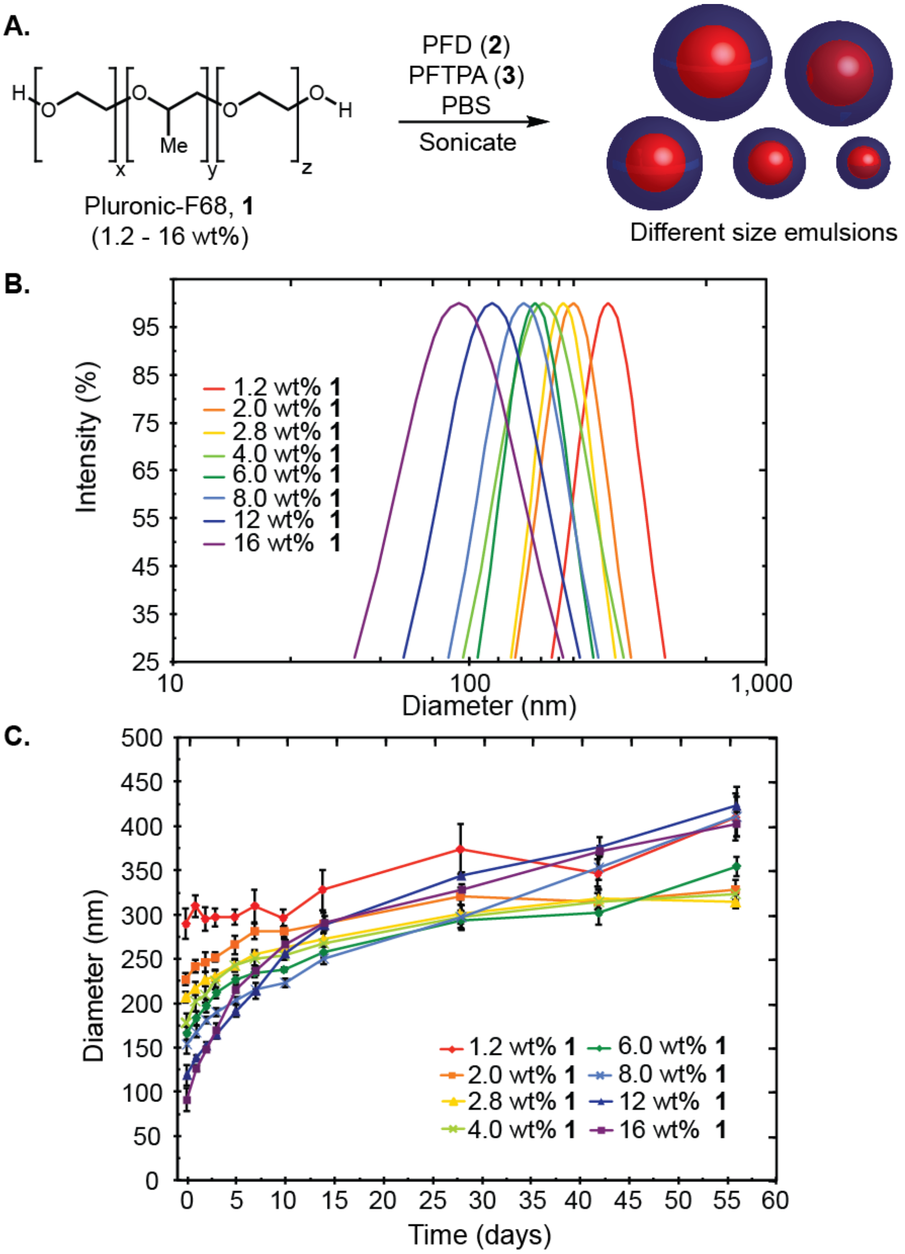

Figure S4. Different size nanoemulsions can be prepared by varying the amount of surfactant. (A) Schematic for Pluronic-F68 nanoemulsion formation. Varying amounts of Pluornic-F68 were dissolved in phosphate buffered saline (PBS) and sonicated in the presence of 7:3 perfluorodecalin (PFD)/perfluorotripropylamine (PFTPA) (20 wt%). (B) Dynamic light scattering (DLS) data for emulsions prepared as described in A. (C) The stability of nanoemulsions prepared in A over two months. The error bars represent the polydispersity of the sample as measured by DLS.

Emulsion Preparation and Characterization

Reagents
Perfluorodecalin (PFD) and perfluorotripropylamine (PFTPA) were purchased from Synquest and used without further purification. Pluronic-F68 was purchased from Sigma-Aldrich. Phosphate buffered saline (PBS) was purchased from Mediatech Inc.

Sonication
Bath sonication was performed in a Branson 3510 sonicator. Probe sonication was performed with a Microson Ultrasonic Cell Disruptor.

Dynamic light scattering and zeta potential
Dynamic light scattering (DLS) data was obtained on a NanoBrook Omni (Brookhaven) instrument. Samples were diluted 1:100 in 0.1x PBS and equilibrated (5 min) to 25 ^{o}C before measurement collection. Light scattering measurements were performed at 90° and size distribution was determined by the NNLS algorithm. Error bars for DLS data represent one polydispersity unit (+/- 0.5 polydispersity) as determined from the average of five DLS experiments (100 s collection each). Zeta potential measurements were obtained on a NanoBrook Omni (Brookhaven) instrument or a Zetasizer Nano ZS (Malvern). Samples were diluted 1:100 in 0.01x PBS and equilibrated (5 min) to 25 ^{o}C before measurement collection. Five replicate measurements (20 scans each) were collected and averaged for all zeta potential data. The Smoluchowski model was employed. Error bars represent two standard deviation units (+/- 1 standard deviation).

Photophysical data
Absorbance spectra were obtained on an Cary 4000 UV/Vis spectrophotometer (Agilent Technologies) with a scan rate of 2000 nm/min. The instrument was blanked on the solvent prior to obtaining a spectrum. Photoluminescence spectra were obtained on a Jobin Yvon/Horiba Instruments spectrophotometer. Absorbance and photoluminescence data were collected in quartz cuvettes.

Confocal Microscopy
Confocal microscopy was performed on a Leica TCS SP2 Confocal Laser Scanning Microscope. All images were acquired with a 63X oil objective. Microscopy samples were prepared on glass slides that were cleaned with acetone (1 h bath sonication), isopropanol (1 h bath sonication) and dried with nitrogen. A "window" of scotch tape was then applied. The sample was dropcast inside the window, a coverslip was placed ontop and secured with clear nail polish.

Infrared spectroscopy
Infrared (IR) spectroscopy was performed on a Thermo Scientific Nicolet 6700 Fourier transform infrared spectrometer using the attenuated total reflectance (ATR) mode on a germanium crystal.

Figure 2
Stock solutions in acetone (10 mM) containing aniline **4**, triazine **5**, trityl **6**, or benzimidazole **7** were prepared. 100, 10, 1, or 0 μL of these stock solutions were added to eppendorf tubes and the acetone was allowed to evaporate overnight. The following morning, PFD (18 μL) and PFTPA (8 μL) were added to each tube and sonicated (bath) until dissolved, at which point 28 mg/mL Pluronic-F68 in PBS (250 μL) was added. Each mixture was sonicated (probe, 0.02 watts) for 15 minutes at 0 ^{o}C. Dynamic light scattering, zeta potential, and absorbance measurements were obtained as described above.

Figure 3
Fifteen different emulsions were prepared in duplicate or triplicate and their size and charge were determined by dynamic light scattering and zeta potential as described above. 28 mg/mL solutions in PBS were prepared for Pluronic-F68 (**1**, Sigma-Aldrich, P1300), poly(β-cyclodextrin) (**8**, Sigma-Aldrich, C2485), poly(acrylic acid) (**9**, Sigma-Aldrich, 181285), poly(styrene sulfonic acid sodium salt) (**10**, Polysciences, 08772), diallyl dimethyl ammonium chloride polymer (**12**, Monomer-Polymer Dajac Laboratories Inc, 8782), poly(allylamine hydrochloride) (**13**, Alfa Aesar, 43092), lignosulfonic acid sodium salt (**14**, Sigma-Aldrich, 370975), Bovine Serum Albumin (**15**, Sigma-Aldrich, A7511), poly(glutamate,tyrosine) sodium salt (**16**, Sigma-Aldrich, P0275), poly-L-(lysine) hydrobromide (**17** Sigma-Aldrich, P7890), Dextran (**18**, Sigma-Aldrich, D1662), Heparin sodium salt (**19**, Akron Biotech, AK3004), and hydrophilic polyphenylene ethylene **20**[1]. For n-type conjugated polymer **21**[2], a 16 mg/mL solution in PBS was prepared. 250 μL of each solution was combined with PFD (18 μL) and PFTPA (8 μL) and sonicated into nanoemulsions (probe, 15 min, 0.02 watts, 0 ^{o}C). For poly(methyl vinyl ether-*alt*-maleic anhydride (**11**, Sigma-Aldrich, 416339) a stock solution (40 mg/mL) in acetone was prepared and 100 μL was placed in an eppendorf tube. Upon evaporation of the acetone, (250μL) was added and sonicated (bath) for ~ 4 hours until enough anhydride had hydrolyzed that the polymer became water-soluble. At this point, PFD (18 μL) and PFTPA (8 μL) were added and the mixture was sonicated (probe, 0.02 watts) for 15 minutes at 0 ^{o}C.

Figure 4B
A stock solution of poly(methyl vinyl ether-*alt*-maleic anhydride) in acetone (40 mg/mL) was prepared and 100 μL was placed in six eppendorf tubes. The acetone was evaporated overnight. The following morning, PBS (200 μL) was added and these mixtures were sonicated (bath) until the solutions were transparent (~4.5 h),

---

[1] VanVeller, B.; Miki, K.; Swager, T.M. "Rigid hydrophilic structures for improved properties of conjugated polymers and nitrotyrosine sensing in water." *Org. Lett.* **2010**, *12*, 1292-1295.
[2] Izuhara, D.; Swager, T.M. "Poly(pyridinium phenylene)s: water-soluble N-type polymers." *J. Am. Chem. Soc.* **2009**, *131*, 17724-17725.

at which point PFD (18 μL) and PFTPA (8 μL) were added. These four samples were then differentiated by the addition of glycine (**22**, 25 μL of 150 mg/mL solution in water plus 25 μL DMSO), methyl glycine (**23**, 50 μL of 126 mg/mL solution in 1:1 DMSO/PBS), methyl arginine (**24**, 50 μL of 261 mg/mL methyl arginine in 1:1 DMSO/PBS), or nothing (50 μL of 1:1 DMSO/PBS). The mixtures were then sonicated (probe, 0.02 watts) for 15 minutes at 0 ^{o}C. The zeta potential of each emulsion was measured as described above. The average of five zeta potential measurements is plotted in Figure 4B.

Figure 4C

The emulsions prepared and analyzed in Figure 4B were dried in a vacuum oven at 40 ^{o}C overnight. The resulting residue was analyzed by ATR-FTIR. Plotted in Figure 4C are the IR spectra from 1800 to 1200 cm^{-1} normalized to the dominant carbonyl stretch in the polymer (1712 cm^{-1}).

Figure 5B
A solution of 28 mg/mL poly(β-cyclodextrin) in PBS (500 μL) was combined with PFD (36 μL) and PFTPA (16 μL) and sonicated (probe, 0.02 watts) for 15 minutes at 0 ^{o}C. The emulsion solution was aliquoted into five portions (50 μL each). A solution of 1-adamantaneamine (**26**, 5 mg/mL) in CH_3CN was prepared. A differing amount of 1-adamantaneamine solution was added to each aliquot (0, 1, 2.5, 5, 10 μL) followed by CH_3CN so that each sample contained 10 μL total CH_3CN. The solutions were mixed, incubated at room temperature for 10 minutes, and the CH_3CN was removed via gentle blowing with nitrogen. The zeta potential of the treated emulsions were measured as described above. The above procedure was repeated with 1-adamantyl carboxylic acid (**25**, 5 mg/mL solution).

Figure 5C+D
A solution of 28 mg/mL poly(β-cyclodextrin) in PBS (750 μL) was combined with PFD (54 μL) and PFTPA (24 μL) and sonicated (probe, 0.02 watts) for 15 minutes at 0 ^{o}C. A solution of 28 mg/mL Pluornic-F68 in PBS (750 μL) was combined with PFD (54 μL) and PFTPA (24 μL) and sonicated (probe, 0.02 watts) for 15 minutes at 0 ^{o}C. This procedure was repeated such that enough perfluorocarbon emulsion solution was obtained. A 1 mg/mL solution of **27** in water was also prepared.

Using the above solutions 6 samples were prepared in triplicate as follows:

(A) 150 μL β-CD emulsions, 50 μL water
(B) 150 μL β-CD emulsions, 25 μL of **27** solution, 25 μL water
(C) 150 μL β-CD emulsions, 50 μL of **27** solution
(D) 150 μL Pluronic-F68 emulsions, 50 μL water
(E) 150 μL Pluronic-F68 emulsions, 25 μL of **27** solution, 25 μL water
(F) 150 μL Pluronic-F68 emulsions, 50 μL of **27** solution
(G) 100 μL β-CD emulsions, 100 μL of **27** solution (Figure 5D)
(H) 100 μL Pluornic-F68 emulsions, 100 μL of **27** solution (Figure 5D)

The solutions were incubated at room temperature for 15 minutes in the dark. The samples were trice washed by centrifugation (3 min at 5000 rpm) and resuspension in PBS (250 μL). Following the third wash, the emulsions were resuspended in PBS (75 μL). The photoluminescence of the resulting perfluorocarbon emulsion solution was measured. Excitation was at 415 nm, emission was collected from 450-650 nm with an integration time of 0.25 s and slit width of 7 nm. Plotted in Figure 5C is the photoluminescence at 520 nm (λ_{max}) for samples A-F.
Samples G and H were diluted 1:10 in PBS and 5 μL was dropcast onto a clean microscope slide. A cover slip was affixed and these samples were analyzed by confocal microscopy. Laser power = 25%, excitation = 458 nm, emission collection 480-600 nm, gain = 721, offset = -24.8, scale bar = 1 micron. No settings were changed in between imaging sample G and H.

Figure 6B-D
Three eppendorf tubes were prepared with rhodamine **28** (0.12 mg) dissolved in PFD (9 μL) and PFTPA (4 μL). To this solution was added 150 μL of (B) 28 mg/mL poly(β-cyclodextrin) in PBS, (C) 14 mg/mL poly(methyl vinyl ether-*alt*-maleic anhydride) in PBS (hydrolyzed by bath sonication for ~4 h), or (D) 28 mg/mL poly(allylamine hydrochloride) in PBS. These mixtures were sonicated (probe, 0.02 watts) for 15 minutes at 0 °C, diluted 1:100 in PBS and 5 μL was dropcast onto slides and imaged as described above.

Figure S1, Figure 3C-H
Emulsions were prepared as described in Figure 3. Dynamic light scattering of each sample was acquired at various timepoints over two months as described above.

Figure S2B
Four solutions of poly(acrylic acid) (**9,** 7mg) in PBS (200 μL) were prepared. These four samples were then differentiated by the addition of glycine (**22**, 25 μL of 150 mg/mL solution in water plus 25 μL DMSO), methyl glycine (**23**, 50 μL of 126 mg/mL solution in 1:1 DMSO/PBS), methyl arginine (**24**, 50 μL of 261 mg/mL methyl arginine in 1:1 DMSO/PBS), or nothing (50 μL of 1:1 DMSO/PBS). The mixtures were then sonicated (probe, 0.02 watts) for 15 minutes at 0 °C. The zeta potential of each emulsion was measured as described above. The average of five zeta pot nail measurements is plotted in Figure S2B.

Figure S2C

The emulsions prepared and analyzed in Figure S2B were dried in a vacuum oven at 40 °C overnight. The resulting residue was analyzed by ATR-FTIR. Plotted in Figure S2C are the IR spectra from 1800 to 1200 cm^{-1} normalized to the dominant carbonyl stretch in the polymer (1712 cm^{-1}).

Figure S3

A solution of 28 mg/mL Pluronic-F68 in PBS (500 μL) was combined with PFD (36 μL) and PFTPA (16 μL) and sonicated (probe, 0.02 watts) for 15 minutes at 0 °C. The nanoemulsion solution was aliquoted into five portions (50 μL each). A solution of 1-adamantaneamine (**26**, 5 mg/mL) in CH_3CN was prepared. A differing amount of 1-adamantaneamine solution was added to each aliquot (0, 1, 2.5, 5, 10 μL) followed by CH_3CN so that each sample contained 10 μL total CH_3CN. The solutions were mixed, incubated at room temperature for 10 minutes, and the CH_3CN was removed via gentle blowing with nitrogen. The zeta potential of the treated nanoemulsions were measured as described above. The above procedure was repeated with 1-adamantyl carboxylic acid (**25**, 5 mg/mL solution).

Figure S4

A stock solution of Pluronic-F68 (80 mg/mL) in PBS was prepared and diluted to 60, 40, 28, 20, and 12 mg/mL with PBS. Additionally, Pluorinc-F68 (30 mg or 40 mg) was dissolved in PBS (250 μL) and sonicated (bath) until dissolved. Each Pluronic-F68 solution (250 μL) was combined with PFD (18 μL) and PFTPA (8 μL) and sonicated (probe) for 15 minutes at 0.02 watts at 0 °C. Dynamic light scattering data was collected as described above.

Synthesis and Characterization of 27

Scheme S1. Synthesis of adamantyl-lucifer yellow **27**.

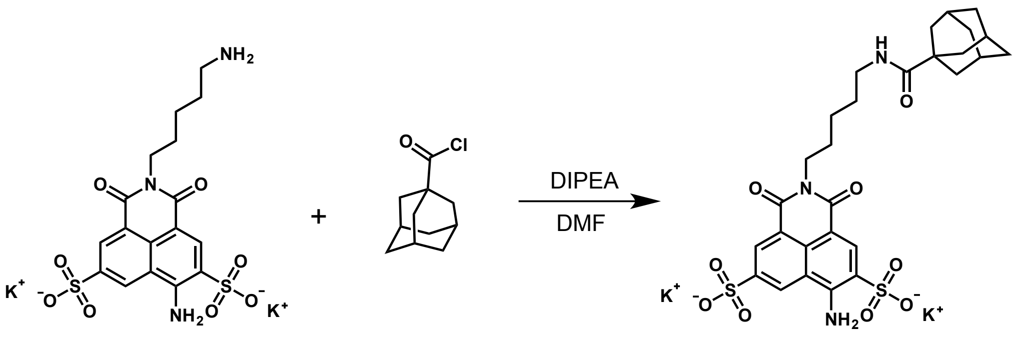

S1 **S2** **27**

General Procedure

Solvent was removed by reduced pressure with an IKA RV-10 Rotovapor equipped with a Welch self-cleaning dry vacuum or house vacuum. Products were further dried by reduced pressure with a Maxima D2A high vacuum. Thin layer chromatography was performed with Baker-flex Silica Gel 1B-F plates (JT Baker). Flash chromatography was performed using technical grade silica gel with 60Å pores and 230-400 mesh particle size (Sigma-Aldrich, 717185). All ^{1}H and ^{13}C spectra are reported in ppm and referenced to solvent peaks. NMR spectra were obtained on Bruker Avance 400 or 600 instruments. High resolution electrospray ionization (ESI) mass spectra were obtained from the MIT Department of Chemistry Instrument Facility.

Lucifer yellow-adamantyl (27). Lucifer yellow cadaverine (Biotium, 10 mg, 0.019 mmol, 1.2 equiv.) was dissolved in DMF (Aldrich, anhydrous, 1 mL) in a flame dried flask. To this solution 1-adamantanecarbonyl chloride (Aldrich, 3 mg, 0.015 mmol, 1 equiv.) and diisopropylethylamine (Aldrich, anhydrous, 10 μL) were added. The mixture was stirred under an Argon atmosphere are room temperature overnight. The following morning the mixture was evaporated to dryness and purified by silica gel chromatography using an ethyl acetate/methanol/water solvent system (100:3:1, 50:3:1, 10:3:1). This procedure resulted in pure **27** (5 mg, 0.007 mmol, 47%) as a yellow solid. ^{1}H NMR (600 MHz, MeOD): δ 9.06 (s, 1H), 8.95 (s, 1H), 8.92 (s, 1H), 7.40 (s, *NH*), 5.20 (s, NH_2), 4.13 (t, J = 6.9 Hz, 2H), 3.18 (q, J = 6.1 Hz, 2H), 1.94 (s, 3H), 1.78 (s, 6H), 1.76-1.70 (m, 6H), 1.68-1.64 (m, 2H), 1.56 (p, J = 7.0 Hz, 2H), 1.39 (p, J = 7.0 Hz, 2H). HRMS (ESI$^-$): Calculated for $C_{28}H_{31}N_3O_9S_2^{-2}$ $[M]^{-2}$: 308.5756; found: 308.5766. Absorbance: below 200 nm (ε > 16000 $M^{-1}cm^{-1}$), 232nm (ε = 10400 $M^{-1}cm^{-1}$), 275 nm (ε = 11400 $M^{-1}cm^{-1}$) 434 nm (ε = 4900 $M^{-1}cm^{-1}$). Emission: 520 nm.

^{1}H-NMR

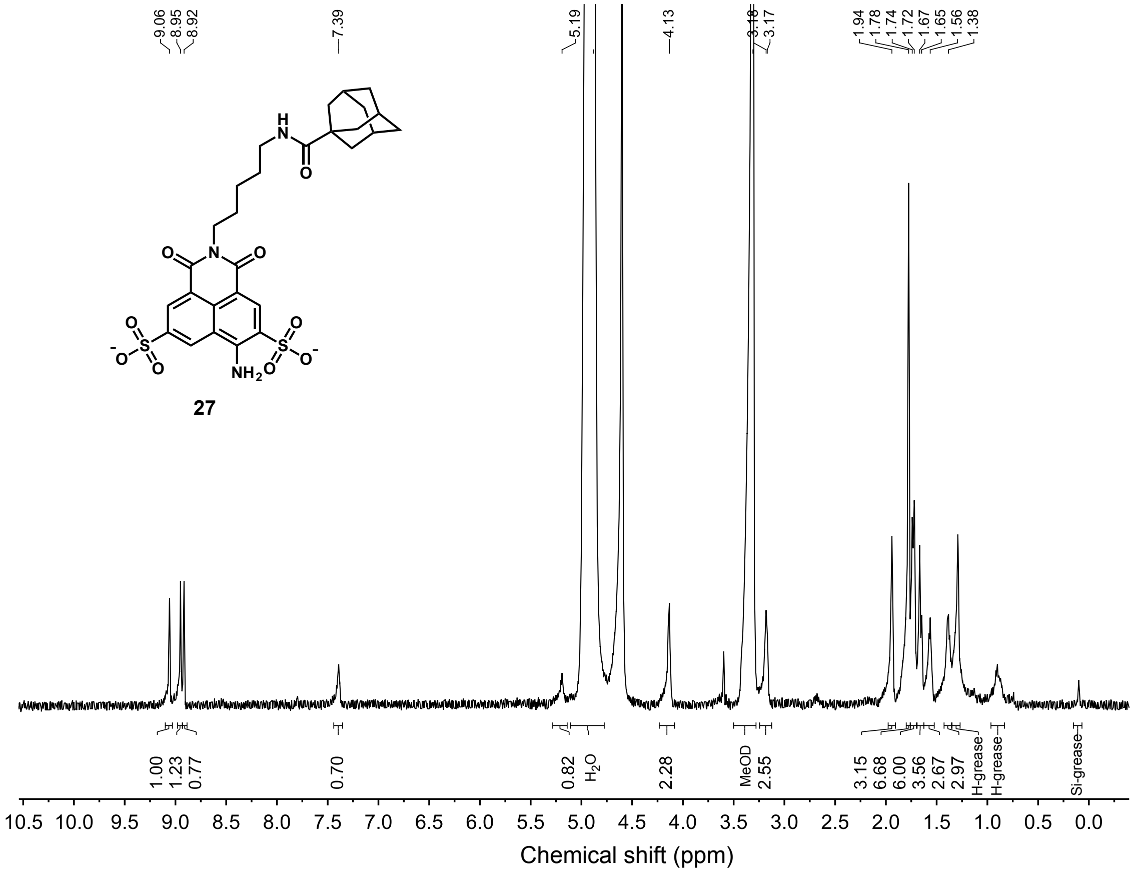

Photophysical Characterization

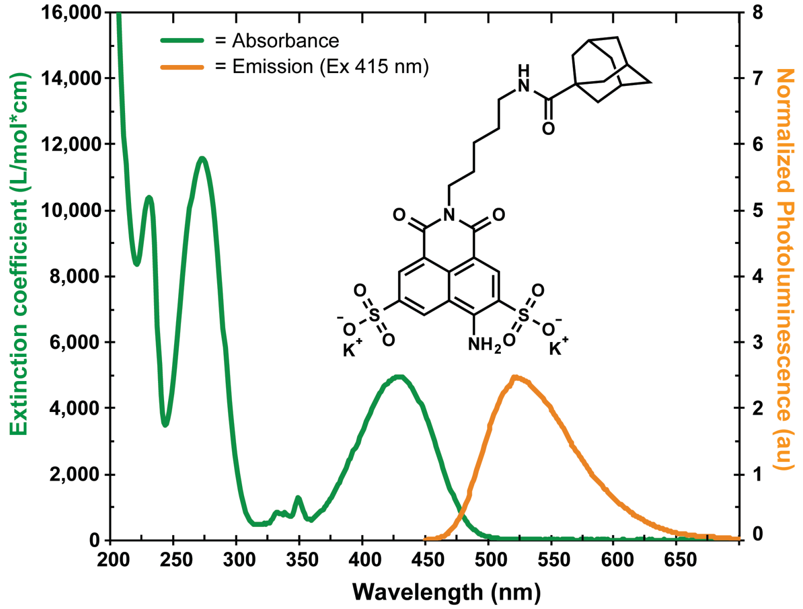

Supplement: Supplementary file 1 [file SC-007-C6SC00341A-s001.pdf]
